# Co-harboring of Novel *bla*_KPC–2_ Plasmid and Integrative and Conjugative Element Carrying Tn*6203* in Multidrug-Resistant *Pseudomonas aeruginosa*

**DOI:** 10.3389/fmicb.2021.674974

**Published:** 2021-07-05

**Authors:** Heng Cai, Yiwei Zhu, Dandan Hu, Yue Li, Sebastian Leptihn, Belinda Loh, Xiaoting Hua, Yunsong Yu

**Affiliations:** ^1^Department of Infectious Diseases, Sir Run Run Shaw Hospital, Zhejiang University School of Medicine, Hangzhou, China; ^2^Key Laboratory of Microbial Technology and Bioinformatics of Zhejiang Province, Hangzhou, China; ^3^Regional Medical Center for National Institute of Respiratory Diseases, Sir Run Run Shaw Hospital, Zhejiang University School of Medicine, Hangzhou, China; ^4^Zhejiang University-University of Edinburgh Institute, Zhejiang University, Haining, China

**Keywords:** plasmid, KPC-2, integrative and conjugative element, prophage, *Pseudomonas aeruginosa*

## Abstract

Many strains of the opportunistic pathogen *Pseudomonas aeruginosa* have acquired resistance to multiple antibiotics. Carbapenem-resistant *P. aeruginosa* poses a global healthcare problem due to limited therapeutic options for the treatment of infections. Plasmids and integrative and conjugative elements (ICEs) are the major vectors of antibiotic-resistance gene transfer. In our study, four carbapenem-resistant strains of *P. aeruginosa* were isolated from the same patient in a tertiary referral hospital in China, one of these was resistant to gentamicin and tobramycin. In this strain P33, we observed a non-transferable plasmid, pP33-2, carrying a novel *bla*_KPC−2_ gene segment (IS*Kpn27*-*bla*_KPC−2_-IS*Kpn6*-*korC*-ORF-*klcA*-IS*26*), which we concluded to have been formed by IS*26*-mediated gene cluster translocation. In addition, by comparing the chromosomes of the *P. aeruginosa* strains that belong to the same sequence type, we identified an ICE, ICEP33, adjacent to a prophage. The *att*L site of ICEP33 is identical to the terminal part of the *att*R site of the prophage. The ICEP33 element contains the transposon Tn*6203*, which encodes antibiotic and metal resistance genes. The insertion of ICEP33 in the chromosome mediates resistance to multiple antibiotics. Our study contributes to the understanding of the acquisition of antibiotic resistance in *P. aeruginosa* facilitated by mobile genetic elements.

## Introduction

Multidrug-resistant (MDR) *Pseudomonas aeruginosa* poses a threat to human health, causing severe acute and chronic infections in hospitalized and immunocompromised patients (Horcajada et al., 2019). Due to the increasing resistance levels globally, the World Health Organization has listed this bacteria as a “critical” pathogen of which research and development for new antibiotics are urgently required (Tacconelli et al., 2018). While carbapenems are currently one of the most important classes of antibiotics for the treatment of MDR *P. aeruginosa* strains, they are ineffective in nearly 30% of clinical cases in China (Hu et al., 2019a).

The acquisition of carbapenemase genes is one of the main mechanisms of resistance to carbapenems (van Duin and Doi, 2017). *Klebsiella pneumoniae* carbapenemases (KPCs), belonging to Ambler’s class A beta-lactamases, have spread extensively and are often horizontally transferred *via* plasmids (Munoz-Price et al., 2013; Chen et al., 2014b). The previous reports have documented KPC-2-producing *P. aeruginosa* ST463 in Hangzhou, China, and typically, the *bla*_KPC−2_ genes were plasmid located (Hu et al., 2015b, 2019b). In addition to intrinsic resistances and chromosomal mutations, mobile genetic elements, such as plasmids and integrative and conjugative elements (ICEs), are responsible for spreading resistance genes to *P. aeruginosa* strains (Botelho et al., 2019).

Integrative and conjugative elements are key players in transmitting antibiotic resistance by conjugation like plasmids and then integrating into the chromosome and being replicated as part of the host genome like bacteriophage (Carraro and Burrus, 2015). They carry certain genes that are required for excision, maintenance, conjugative transfer, and integration into the host genome. ICEs may also carry cargo genes that confer diverse phenotypes on the organisms including pathogenicity islands containing virulence-associated genes (Wurdemann and Tummler, 2007; Kung et al., 2010; Roy Chowdhury et al., 2017).

In this study, we found a clinical *P. aeruginosa* isolate carrying a novel *bla*_KPC−2_ plasmid, pP33-2, markedly different from the *bla*_KPC−2_ carrying plasmids reported previously. This strain also harbored an ICE, ICEP33 containing a Tn*6203* transposon with several resistance genes.

## Materials and Methods

### Clinical Isolates and Identification

Four isolates, P20, P22, P23, and P33, were cultured from a patient’s rectal and throat swabs and tracheotomy tube using Pseudomonas Isolation Agar (Peptone 20 g/l, MgCl_2_ 1.4 g/l, K_2_SO_4_ 10 g/l, Triclosan 25 mg/l, Agar 13.6 g/l, and Glycerol 20 ml/l.). Bacterial species were identified by the matrix-assisted laser desorption ionization-time of flight mass spectrometry (MALDI-TOF MS; Bruker Daltonik GmbH, Bremen, Germany) and 16S rRNA gene sequencing.

### Antimicrobial Susceptibility Testing

Antimicrobial susceptibility testing was performed by broth microdilution. Antibiotics included: piperacillin; piperacillin-tazobactam; ceftazidime; ceftazidime-avibactam; cefepime; aztreonam; imipenem; meropenem; gentamicin; tobramycin; amikacin; ciprofloxacin; levofloxacin; and colistin. The results were interpreted according to Clinical and Laboratory Standards Institute guidelines (CLSI; M100, 30th Edn.; CLSI, 2020). *P. aeruginosa* strain ATCC 27853 was used as the quality control.

### Genome Sequencing and Analysis

Genomic DNA was extracted by the QIAamp DNA Mini Kit (Qiagen, Germany) and sequenced by HiSeq (Illumina, San Diego, CA, United States). The paired-end reads were assembled by CLC genomics workbench (version 12). Multilocus sequence typing was identified by the PubMLST^1^ (Jolley et al., 2018). Antibiotic resistance genes were identified by the ResFinder 4.1^2^ (Zankari et al., 2012) and were further confirmed by PCR. The genomic DNA of P23 and P33 was additionally prepared by the Gentra Puregene Yeast/Bact. Kit (Qiagen) and Nanopore sequencing was performed by a MinION sequencer (Oxford Nanopore Technologies, Oxford, United Kingdom). *De novo* assembly of the Illumina and Nanopore reads was performed using the Unicycler v0.4.8 (Wick et al., 2017). The genome sequence was annotated by Prokka^3^ (Seemann, 2014) and manually refined by NCBI Blast (Camacho et al., 2009). The nucleotide polymorphism (SNP) tree was built by snippy-multi and FastTree,^4^ visualized by ggtree v2.0.4 (Yu, 2020). Conjugative apparatus was predicted by oriTfinder^5^ and MOBscan (Li et al., 2018; Garcillan-Barcia et al., 2020). ICEs were predicted by ICEfinder^6^ (Liu et al., 2019).

### Conjugation

Conjugation assays were conducted using rifampicin-resistant *Escherichia coli* EC600 and a spontaneous rifampicin-resistant mutant of *P. aeruginosa* PAO1 as the recipient strain. Strain P33 was used as the donor. The selective Mueller-Hinton agar plates contained rifampicin (300 μg/ml) combined with meropenem (0.5 μg/ml) or meropenem (4 μg/ml). Each conjugation experiment was repeated at least three times.

### Accession Numbers

The complete sequences of the chromosomes and plasmids from *P. aeruginosa* strain P23 and P33 have been deposited in the GenBank nucleotide database under accession numbers CP065417-CP065418 and CP065412-CP065416, respectively. The genome sequences of *P. aeruginosa* strains P20 and P22 have been deposited in the GenBank database under accession numbers JAEMVM000000000 and JAEMVL000000000, respectively.

## Results and Discussion

### Case Report

In August 2019, a 69-year-old man was hospitalized in Sir Run Run Shaw Hospital (Mayo Clinic Care Network) in Hangzhou, China. The patient had been suffering from a severe cough with expectoration for 5 months, in addition to fever and hypotension that began approximately 1 month before hospitalization. The patient’s medical history included hypertension, cerebral infarction, and multiple pulmonary infections. Several months before hospitalization, the patient suffered from a bloodstream infection by *P. aeruginosa*. After this renewed hospitalization, the patient’s symptoms were controlled by antibiotics and other supportive treatment, such as mechanical ventilation and fluid therapy. The course of antibiotics used is listed in chronological order as follows: cefoperazone-sulbactam, cefuroxime, piperacillin-tazobactam, and cefuroxime. After 1 month, his condition improved and he was discharged. Four *P. aeruginosa* strains P20, P22, P23, and P33 were isolated from different anatomical locations or at different time points during hospitalization (Table 1). Specifically, P20 was isolated from the rectal swab on the day of admission; P22 and P23 were isolated from the rectal swab and throat swab on the fifth day after admission, respectively; and P33 was isolated from the tracheotomy tube on the twelfth day.

**Table 1:** Summary of antimicrobial susceptibility testing for four strains.

	Antibiotics	P20 (rectal swab, day 0)	P22 (rectal swab, day 5)	P23 (throat swab, day 5)	P33 (tracheotomy tube, day 12)
MIC (μg/ml)	Piperacillin	>256 (R)	>256 (R)	>256 (R)	>256 (R)
	Piperacillin-tazobactam	>256/4 (R)	>256/4 (R)	>256/4 (R)	>256/4 (R)
	Ceftazidime-avibactam	32/4 (R)	16/4 (R)	16/4 (R)	16/4 (R)
	Ceftazidime	256 (R)	256 (R)	256 (R)	128 (R)
	Cefepime	>256 (R)	>256 (R)	>256 (R)	>256 (R)
	Aztreonam	>128 (R)	>128 (R)	>128 (R)	>128 (R)
	Imipenem	>128 (R)	>128 (R)	>128 (R)	>128 (R)
	Meropenem	>128 (R)	>128 (R)	>128 (R)	>128 (R)
	Colistin	0.5 (I)	0.5 (I)	0.5 (I)	0.5 (I)
	Gentamicin	1 (S)	1 (S)	1 (S)	>64 (R)
	Tobramycin	0.5 (S)	0.5 (S)	0.5 (S)	>64 (R)
	Amikacin	4 (S)	4 (S)	4 (S)	8 (S)
	Ciprofloxacin	16 (R)	16 (R)	16 (R)	16 (R)
	Levofloxacin	>64 (R)	>64 (R)	>64 (R)	>64 (R)

*S, sensitive; I, intermediate; and R, resistant*.

### Results of Antimicrobial Susceptibility Testing

The four *P. aeruginosa* isolates were all resistant to penicillins, β-lactam/β-lactamase inhibitor combination agents, cephems, monobactams, carbapenems, and fluoroquinolones and intermediate to colistin. P20, P22, and P23 were susceptible to aminoglycosides while P33 was susceptible to amikacin but resistant to gentamicin and tobramycin (Table 1).

### Genetic Relationship and Antibiotic Resistance Genes of the Strains

**Figure 1:**
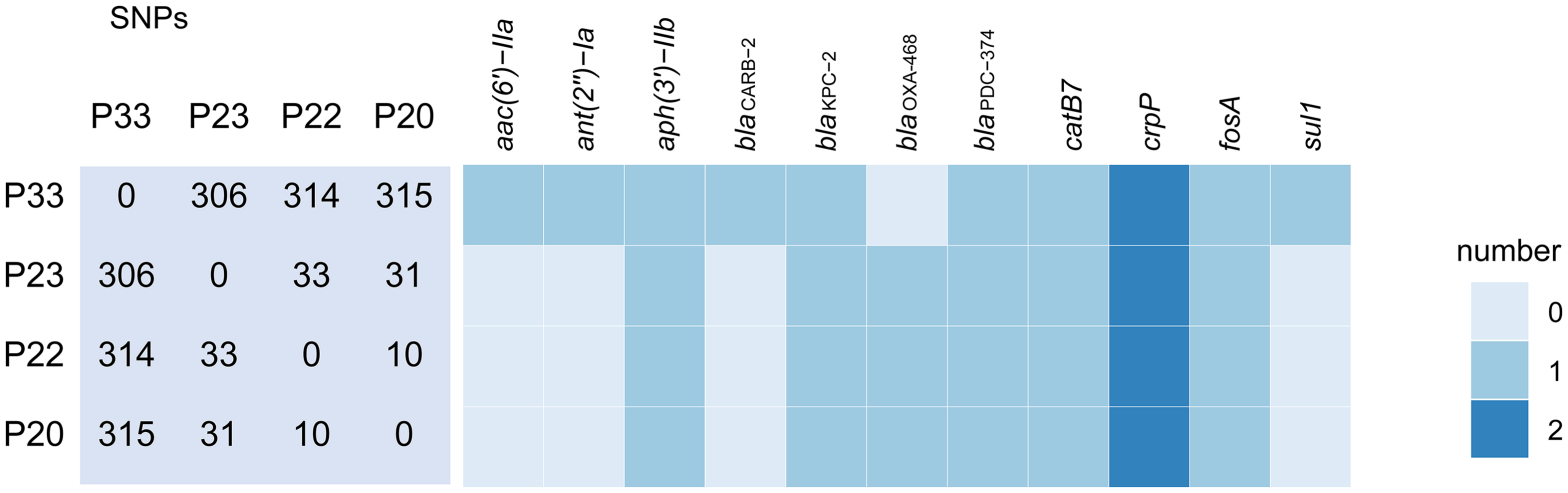
SNP difference matrix and heat map of predicted antibiotic resistance genes of four *Pseudomonas aeruginosa* strains. The numbers in the matrix are the number of variant SNPs between the strains. The indicator denotes the cope number of genes by different color.

P20, P22, P23, and P33 were all identified as ST463. An analysis based on SNP showed that P33 was more genetically distant from the other three strains (Figure 1). P33 differed from P20 in 315 SNPs, from P22 in 314 SNPs, and from P23 in 306 SNPs. Taking the large number of varied SNPs into consideration, it seems that P33 unlikely evolved from the other three strains recently, i.e., during the hospitalization.

The antimicrobial resistance genes of P33 were different from the other isolates. The genome of P33 contained genes that conferred resistance to β-lactams (*bla*_CARB-2_), aminoglycosides (*aac(6')−IIa*, *ant(2")−Ia*), and sulfonamide (*sul1*), which were absent in P20, P22, and P23. While P33 lacked *bla*_OXA-468_, the other three strains contained the gene. All of four strains had *aph(3')−IIb*, *bla*_KPC−2_, *bla*_PDC−374_, *catB7*, *crpP*, and *fosA* (Figure 1). The genes *aac(6')−IIa* and *ant(2")−Ia* are likely to confer the resistance in P33 to gentamicin and tobramycin. Nanopore sequencing and subsequent analysis showed that the carbapenem-resistant gene *bla*_KPC–2_ is located in a plasmid found in P23 and P33, respectively. Sequence analysis indicated that P20, P22, and P23 carry a closely related *bla*_KPC−2_ plasmid, while the one in the P33 is different. The nucleotide sequence of the *bla*_KPC−2_ plasmid in P33 is about in 30% identical to those in others.

The observations of our study reveal differences in SNP and antimicrobial susceptibility among the strains of the same sequence type (ST463). We speculate that the P33 strain could have acquired the mobile elements carrying the relevant antibiotic resistance genes by exchanging genes with other bacteria in the host microbiome during the long-term repeated infection.

### Characterization of Plasmids in P33

Employing Nanopore sequencing, we were able to identify four plasmids in P33, named pP33-1, pP33-2, pP33-3, and pP33-4. Plasmid pP33-1 is 49,654 bp long with an average GC content of 59% and 58 predicted open reading frames (ORFs). Plasmid pP33-2 has a length of 48,306 bp, with an average GC content of 59%. Sequence annotation predicted 64 ORFs. Plasmid pP33-3 is smaller with 3,014 bp, an average GC content of 61% and only three predicted ORFs. Finally, pP33-4 was predicted to contain three ORFs on a similarly sized plasmid (2,953 bp) with an average GC content of 65%.

The genetic structure of pP33-2 can be divided into the general maintenance and the *bla*_KPC−2_ segment. The general maintenance includes the replication initiation (*repA*), partitioning (*parAB*), and genes for plasmid maintenance. The plasmid pP33-2 is classified as an unidentified incompatibility (Inc.) type. BLAST analysis showed that pP33-2 is similar to plasmid ID40_1 (LR700249.1), pPA7790 (CP015000.1), pPB353_1 (CP025052.1), pPB354_1 (CP025054.1), pMS14403A (CP049162.1), pJUPA4295 (LC586269.1), and an unnamed plasmid in strain AR_0230 (CP027175.1), with query coverage ranging from 59 to 78% and an identity of 95.24–98.51%. After removing the *bla*_KPC−2_ cluster, pP33-2 shares 97–100% coverage with the plasmid sequences mentioned above. Thus, while these plasmids share similar backbones, only pP33-2 carries the *bla*_KPC−2_ gene, indicating that pP33-2 is a novel *bla*_KPC−2_ plasmid in *P. aeruginosa*.

Plasmid pP33-1 encodes four components of a conjugative apparatus: an origin of transfer (oriT), a relaxase (R), a type IV coupling protein (T4CP), and a type IV secretion system (T4SS; Smillie et al., 2010), while pP33-2 was predicted to harbor only a MOB_C_ family relaxase and part of T4SS. Although pP33-1 was included as a conjugative helper, conjugation experiments with pP33-2 failed several times, suggesting that pP33-2 might be not transferable.

### Evolution of the *bla*_KPC–2_ Gene Cluster Within the Strains

**Figure 2:**
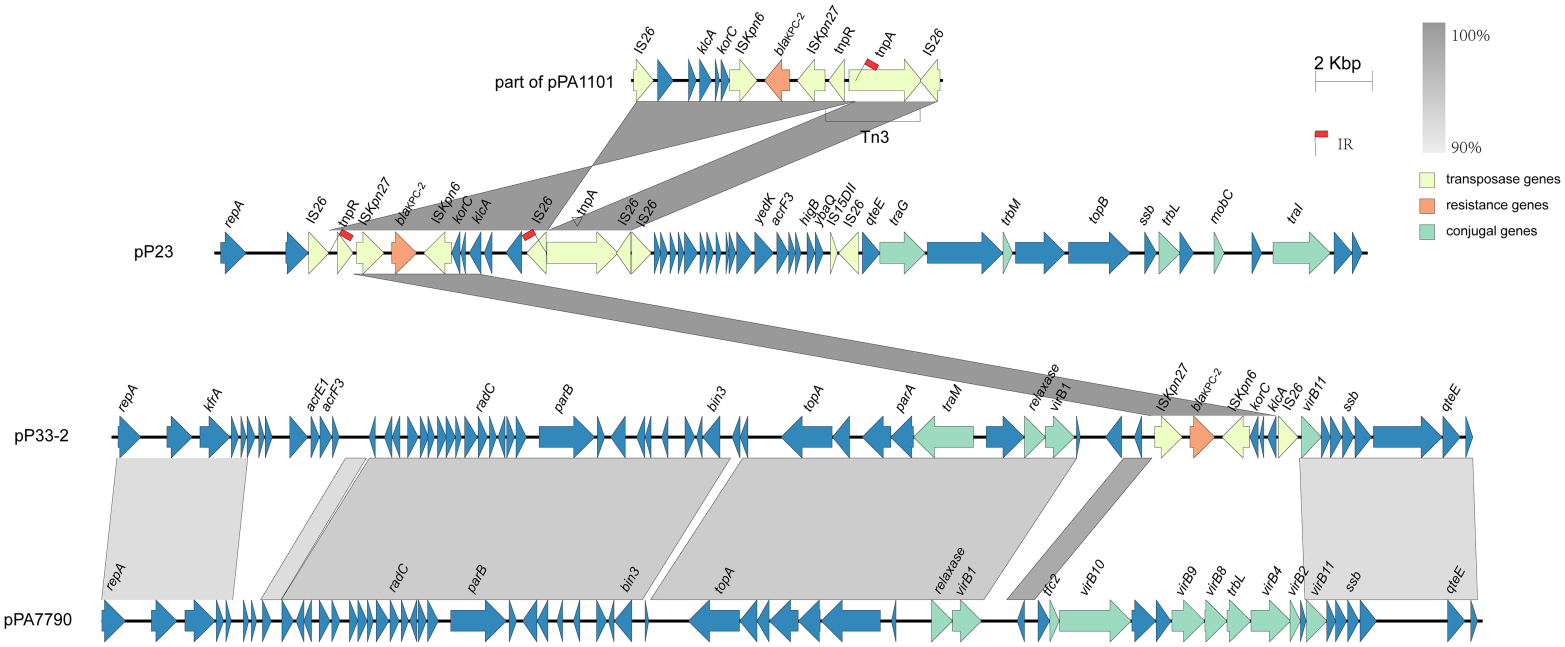
Detailed structure and comparison of the *bla*_KPC–2_ gene cluster within the strains. Yellow, orange, and green arrows represent transposase, resistance, and conjugal genes, respectively. Arrows indicate the translation orientation of the coding genes. Red flags highlight the positions of inverted repeats.

Strain P23 harbors the *bla*_KPC−2_ gene-containing plasmid pP23, which is very similar to plasmid YLH6_p3 (MK882885.1) and pPA1101 (MH734334.1; Hu et al., 2019b). The accessory modules of *bla*_KPC−2_ were different in pPA1101, pP23, and pP33-2 (Figure 2). In pPA1101, there was a core module Tn3-IS*Kpn27*-*bla*_KPC−2_-IS*Kpn6*-*korC*-ORF-*klcA*-ORF-ORF with two inverted IS*26* elements at both ends. The core module Tn3-IS*Kpn27*-*bla*_KPC–2_-IS*Kpn6* was frequently identified as the *bla*_KPC–2_ gene cluster in China (Shen et al., 2009; Cai et al., 2014; Chen et al., 2014a; Li et al., 2015). Occasionally, the core module was observed to be interrupted by the IS*26*-based composite transposons (Chen et al., 2014c). When comparing pP23 to pPA1101, a part of the core module appeared to be reversed (Figure 2). Due to the intramolecular replicative transposition in inverse orientation, the segment between the original IS*26* and the targeted site on *tnp*A was reversed. An indication that supports our interpretation is that we observed 8-bp sequences (5'-CGATATTT-3') reverse complements of each other that flank the two IS*26* copies (Harmer et al., 2014; He et al., 2015; Partridge et al., 2018).

Unlike sequences reported previously, IS*Kpn27*-*bla*_KPC−2_-IS*Kpn6*-*korC*-ORF-*klcA*-IS*26* was found in the KPC module of pP33-2. The gene cluster *korC*-ORF*-klcA* is commonly connected with the core module IS*Kpn27*-*bla*_KPC−2_-IS*Kpn6* as mentioned above (Shen et al., 2009; Dai et al., 2016; Shi et al., 2018). However, unilateral IS*26* element insertion is rarely observed. A possible explanation for this is if IS*26* and the adjacent region that included IS*Kpn27*-*bla*_KPC−2_-IS*Kpn6*-*korC*-ORF-*klcA*, formed a transferable unit (TU). This TU might then have inserted into another plasmid. Normally, the integration results in two copies IS*26* in direct orientation, one at each boundary between the two participating molecules (Harmer et al., 2014; Harmer and Hall, 2015; Varani et al., 2021). However, we observe only one IS*26* in pP33-2; the other might possibly have been lost through a deletion event.

Blast search results identified plasmid pPA7790 (Nascimento et al., 2016), which was isolated from a patient in Brazil, as being most similar to pP33-2 with 78% query coverage and 98.51% identity. Comparing pP33-2 to pPA7790, the backbone is similar, yet the *bla*_KPC–2_ segment in pP33-2 substituted a part of T4SS module in pPA7790 (Figure 2).

The mechanism of how the *bla*_KPC−2_-carrying plasmid originated has not been completely established. However, it is very plausible that the IS*26* facilitated the spread of KPC among different plasmids in *P. aeruginosa*. The *P. aeruginosa* strain with novel *bla*_KPC−2_ plasmid described here might become an important regional epidemic clone.

### Integration of ICEP33 Brings Resistance Genes

**Figure 3:**
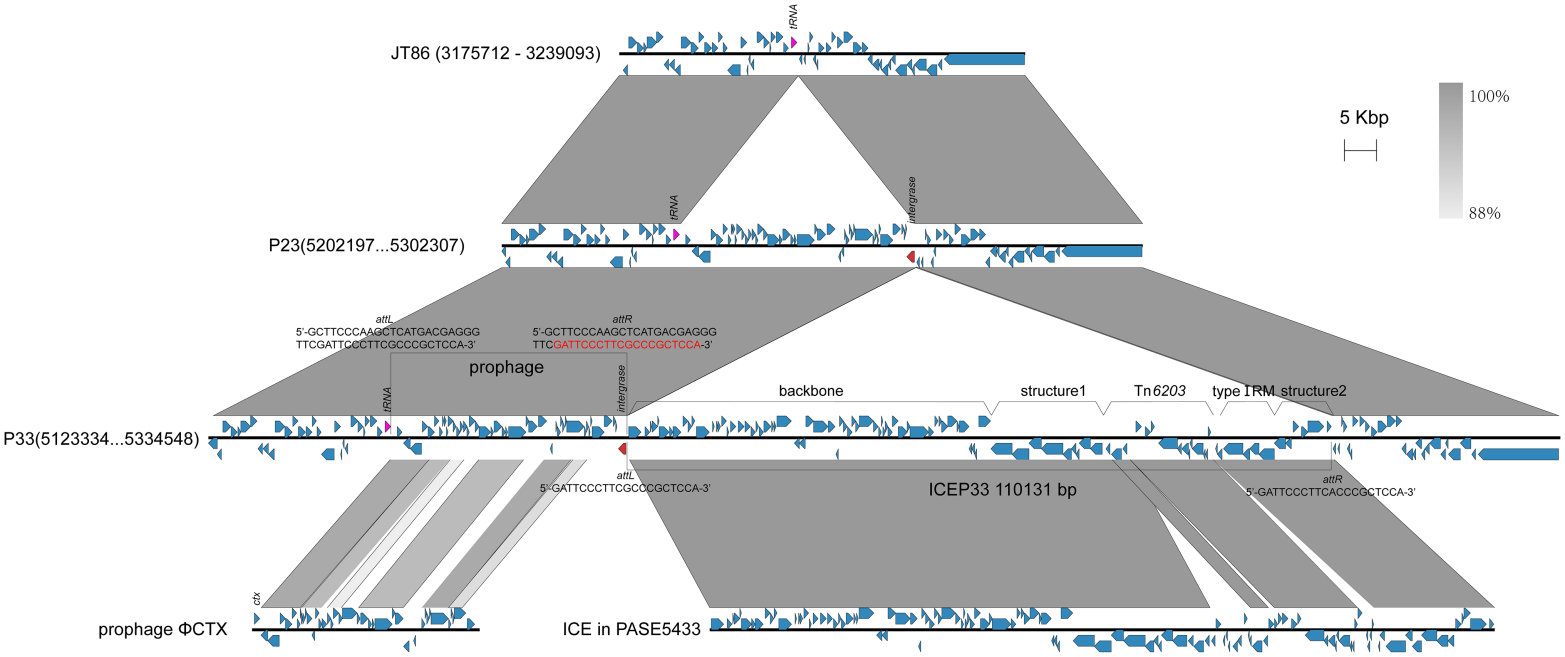
Comparison of partial chromosomes. A prophage integrated into the chromosome first, and then an ICE integrated next to the prophage. The *att*L and *att*R of prophage and ICE are, respectively, labeled. The overlapping att site is marked red. Red arrows represent the genes which ICE and prophage integrate adjacent to. The ICE is divided into four parts.

Among the four clinical isolates, P23 and P33 have the closest genetic relationship (Figure 1). A comparison of the chromosomes of P23 and P33 showed there is an 110,131-bp-long insertion downstream of the gene encoding the integrase family protein on the P33 chromosome (Figure 3). ICEfinder predicted it to be a putative ICE, hereafter named ICEP33. Within this element, we found the aminoglycosides-resistant genes *aac(6')-IIa* and *ant(2")-Ia* together with *bla*_CARB-2_ and *sul1*, which were only found in P33.

The antimicrobial resistance genes are organized within a single class I integron integrated in transposon Tn*6203*. In addition, there is a Tn*4378*-like transposon carrying a mercury resistance operon (*mer*) in Tn*6203*. With the exception of the resistance segment, ICEP33 could roughly be divided into four parts: backbone, structure 1, type I restriction and modification (RM) system, and structure 2 (Figure 3). The backbone contains genes encoding a bacteriophage P4-like integrase, conjugative apparatuses, and genes for DNA processing and replication. The type I RM is possibly required for ICE self-maintenance (Hu et al., 2015a). Structure 1 and structure 2 contain genes encoding proteins, such as cold shock domain-containing protein, S8 family peptidase and LysR family transcriptional regulator. These structures might be acquired through multiple recombination events during the ICE evolution.

The insertion of ICE is flanked by two attachment (*att*) sites at both ends of the sequence (*att*L and *att*R; Bellanger et al., 2011; Johnson and Grossman, 2015). In P33, the *att*L is 5'-GATTCCCTTCGCCCGCTCCA-3' and the *att*R is 5'-GATTCCCTTCACCCGCTCCA-3'. These site-specific integrations mediated by the bacteriophage P4-like integrase are typically found within the tRNA gene (Ravatn et al., 1998a,b; Wozniak and Waldor, 2010; Johnson and Grossman, 2015). However, ICEP33 is integrated approximately 37-kb downstream of tRNA^Gly^. We found an intact prophage sequence inserted next to the tRNA^Gly^ causing 46 bp direct repeats, which contain the *att* site of ICEP33 (Figure 3). This prophage is 36.7 kb in length with an average GC content of 63.8% and 48 predicted ORFs. The prophage encodes 42 phage proteins and 33 of them are similar to P2-like phage ΦCTX (AB008550.1). Compared to ΦCTX, the prophage in P33 lacks the cytotoxin gene (*ctx*) and contains a different integrase gene. We propose that the evolution of this genetic section of the bacterial chromosome happened in succession, with integration of the prophage into the chromosome similar to JT86 (CP062219.1) occurring first, forming the genomic organization that is also observed in P23. In a second step, an ICE integrated next to the prophage and eventually leading to the structure observed in P33 (Figure 3). ICEs with bacteriophage P4-like integrase are often found adjacent to phages that target the same *att* site (Botelho et al., 2018). Our study provides a possible order of their insertion. However, whether there is an interaction in integration between ICE and prophage is unclear.

### ICEP33 Belongs to *clc*-Like Family

**Figure 4:**
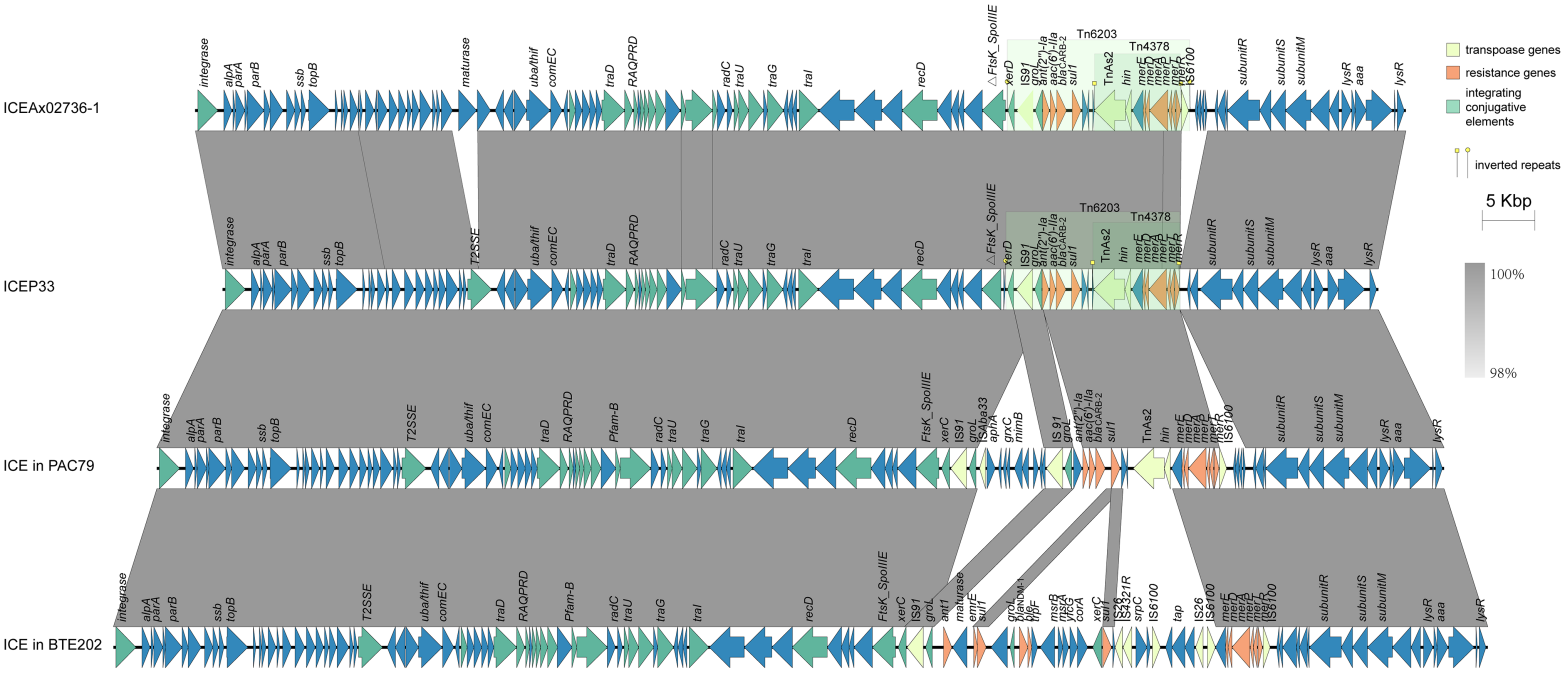
Detailed structure and comparison of similar ICEs. Yellow, orange, and green arrows represent transposase genes, resistance genes, and integrating conjugative elements, respectively. Arrows indicate the translation orientation of the coding genes. Two green shades denote Tn*6203* and Tn*4378*.

Two large families of ICEs exist in *P. aeruginosa*, the pKLC102 family and the *clc*-like family, which includes *clc* (AJ617740), PAGI-2 (AF440523), and PAGI-3 (AF440524; Kung et al., 2010). ICEP33 is similar to PAGI-15 (KX196168.1) and PAGI-16 (KX196169.1), which were defined as *clc*-like ICEs (Hong et al., 2016), indicating that ICEP33 belongs to the same family. These ICEs all share a similar bipartite structure: the conserved part adjacent to the integration site as backbone and the variable part that consisted of unique cargo ORFs (Kung et al., 2010). ICEP33-like ICEs were also found in other bacteria, such as *Achromobacter xylosoxidans* strain X02736 (GCA_000517225.1; Hu et al., 2015a), *Bordetella trematum* strain E202 (CP049957.1), *P. aeruginosa* strain SE5443 (CP046405.1), and C79 (CP040684.1; Figures 3, 4). The antibiotic resistance genetic load is the main difference between them. Compared to the most similar sequence ICEAx02736-1, they both carry the same resistance genes embedded in Tn*6203* modules. In P33, the right end of Tn*6203* was truncated by IS6100 and four predicted ORFs, which led to the loss of an inverted or direct repeat. In addition, it appears that a gene encoding group II intron reverse transcriptase/maturase had inserted into the middle of the gene encoding a DEAD/DEAH box helicase. Interestingly, these ICEs in Figures 3, 4 are found in different bacterial species in China, indicating that this type of ICE is present in a variety of hosts and may be an important molecular mechanism for the dissemination of resistance genes in the country.

## Conclusion

In this study, four *P. aeruginosa* strains were isolated from a single patient in an intensive care unit. The horizontal transmission of the antibiotic resistance genes in those strains was hypothesized to be mediated by mobile elements IS*26* and ICEs. In this work, a novel plasmid pP33-2 encoding *bla*_KPC–2_ is described, which likely originates from an IS*26*-mediated insertion into the position of T4SS; subsequently, a deletion of the IS*26* and a partial removal of the T4SS occurred during evolution. In addition, the resistance genes embedded in the transposon Tn*6203* became part of ICEP33 that was found in the same strain. ICEP33, which belongs to *clc*-like family is integrated adjacent to a prophage sequence. The appearance of the novel *bla*_KPC−2_ plasmid and the integration of the ICE has created a new type of MDR *P. aeruginosa* strain, which creates a high risk of a regional epidemic carbapenem-resistant clone, bringing more challenges to the clinical treatment of infections caused by *P. aeruginosa*.

## Data Availability Statement

The datasets presented in this study can be found in online repositories. The names of the repository/repositories and accession number(s) can be found at: https://www.ncbi.nlm.nih.gov/genbank/ (CP065417-CP065418, CP065412-CP065416, JAEMVM000000000, and JAEMVL000000000).

## Author Contributions

XH, YY, and HC designed the study. HC, YZ, DH, and YL performed the experiments. HC and YZ analyzed the data. HC drafted the article. XH, YY, SL, and BL revised the article. All authors have approved the final article.

### Conflict of Interest

The authors declare that the research was conducted in the absence of any commercial or financial relationships that could be construed as a potential conflict of interest.

## Footnotes

^1^https://pubmlst.org/organisms/pseudomonas-aeruginosa

^2^https://cge.cbs.dtu.dk/services/ResFinder/

^3^https://github.com/tseemann/prokka

^4^https://github.com/tseemann/snippy

^5^https://tool-mml.sjtu.edu.cn/oriTfinder

^6^https://db-mml.sjtu.edu.cn/ICEfinder
