# Supplementary material for: Co-harboring of Novel blaKPC–2 Plasmid and Integrative and Conjugative Element Carrying Tn6203 in Multidrug-Resistant Pseudomonas aeruginosa
Source: Front Microbiol. 2021 Jul 5;12:674974. doi: 10.3389/fmicb.2021.674974 (PMC8287167; doi:10.3389/fmicb.2021.674974)
Supplement: Supplementary file 1 [file Data_Sheet_1.PDF]

Table S1.1 P23-unicycler

Segment	Length	Depth	Starting gene	Position	Strand	Identity	Coverage
1	6,909,254	1.00x	UniRef90_B7V0N6	170,127	reverse	99.80%	100.00%
2	40,937	1.42x	UniRef90_G3JZU7	16,846	forward	100.00%	100.00%

Table S1.2 P33-unicycler

Segment	Length	Depth	Starting gene	Position	Strand	Identity	Coverage
1	7,013,659	1.00x	UniRef90_B7V0N6	5,355,808	reverse	99.80%	100.00%
2	49,654	1.44x	none found				
3	48,306	0.49x	none found				
4	3,014	26.29x	none found				
5	2,953	34.61x	none found				

Table S2 pP33-2 annotation by HMMER results summary

Sequence Number	Query Name	Hits Found	Status	Top Hit			Details
				Identifier	Description	E-value	
1	I5U70_33045	4	DONE	A0A1H3ZMY8_9FIRM	Uncharacterized protein, contains FMN-binding domain	0.0021	show
2	I5U70_33050	5	DONE	A0A1H0PWP9_9CLOT	Ribosomal protein L7/L12 C-terminal domain-containing protein	6.60E-06	show
3	I5U70_33055	367	DONE	A0A1M5E3D9_9GAMM	Plasmid stabilization system protein ParE	2.70E-47	show
4	I5U70_33060	415	DONE	B2VGM3_ERWT9	RHH_1 domain-containing protein	2.10E-44	show
5	I5U70_33065	5	DONE	A0A1H3NA13_9ACTN	Uncharacterized protein	4.20E-06	show
6	I5U70_33070	1	DONE	A0A1S3IP68_LINUN	adipocyte plasma membrane-associated protein	0.0073	show
8	I5U70_33075	18	DONE	A0A010RP49_PSEFL	KORA domain-containing protein	2.10E-21	show
11	I5U70_33080	7157	DONE	A0A2N5XZ41_9GAMM	DNA repair protein	9.60E-44	show
13	I5U70_33085	0	DONE				show
14	I5U70_33090	22	DONE	A0A1L6I311_9BURK	Uncharacterized protein	6.00E-08	show
15	I5U70_33095	2	DONE	A0A2C9L0S0_BIOGL	Uncharacterized protein	0.00016	show
16	I5U70_33100	0	DONE				show
17	I5U70_33105	4797	DONE	A0A366H4K4_9BURK	ParB family chromosome partitioning protein	6.60E-150	show
18	I5U70_33110	20	DONE	A0A010SZF4_PSEFL	Uncharacterized protein	1.10E-36	show
19	I5U70_33115	3	DONE	S6BRA9_PSERE	Uncharacterized protein	5.00E-80	show
20	I5U70_33120	238	DONE	A0A1V0B6H0_9PSED	Uncharacterized protein	2.80E-21	show
21	I5U70_33125	1	DONE	D1P8D8_9GAMM	Cadherin_4 domain-containing protein (Fragment)	0.00014	show

22	I5U70_33130	7	DONE	W1J9I2_9GAMM	Uncharacterized protein	9.60E-14	show
23	I5U70_33135	1421	DONE	A0A162MTJ5_9FIRM	HTH-type transcriptional regulator Xre	1.40E-17	show
24	I5U70_33140	2	DONE	T1I626_RHOPR	Uncharacterized protein	0.00017	show
25	I5U70_33145	0	DONE				show
26	I5U70_33150	1	DONE	A0A098G0I3_9GAMM	ANK_REP_REGION domain-containing protein	3.30E-09	show
27	I5U70_33155	3818	DONE	A0A0D1PNZ1_PSEPU	Serine recombinase	1.20E-90	show
28	I5U70_33160	2	DONE	A0A1D6MKM9_MAIZE	S-adenosyl-L-methionine-dependent methyltransferase superfamily protein	0.0064	show
29	I5U70_33165	26	DONE	A0A2T6FPQ0_9GAMM	Uncharacterized protein	1.40E-08	show
30	I5U70_33170	0	DONE				show
31	I5U70_33175	13294	DONE	G7QB84_9DELT	DNA topoisomerase 1	5.20E-151	show
32	I5U70_33180	1	DONE	N6X7J7_9ALTE	Uncharacterized protein	8.10E-28	show
33	I5U70_33185	4	DONE	A0A2Z4V8E7_9ACTN	Uncharacterized protein	6.30E-11	show
34	I5U70_33190	311	DONE	N6W3K8_9ALTE	ParB domain-containing protein	1.20E-61	show
35	I5U70_33195	12787	DONE	A0A399T2U1_9BACT	ParA family protein	7.30E-22	show
36	I5U70_33200	78	DONE	N6X121_9ALTE	TraG-D_C domain-containing protein	7.10E-145	show
38	I5U70_33205	18	DONE	N6X7K3_9ALTE	Uncharacterized protein	2.80E-71	show
39	I5U70_33210	21144	DONE	N6WA66_9ALTE	CpaF family protein	1.90E-102	show
40	I5U70_33215	214	DONE	A0A3D8PCI4_9RHOB	P-type conjugative transfer ATPase TrbB	2.30E-05	show
41	I5U70_33220	42	DONE	A0A101M811_9EURO	WD_REPEATS_REGION domain-containing protein	3.50E-13	show
42	I5U70_33225	38	DONE	Q6LSH8_PHOPR	Uncharacterized protein	2.70E-40	show

44	I5U70_33230	1	DONE	N6W3M0_9ALTE	Uncharacterized protein	1.90E-12	show
46	I5U70_33235	3930	DONE	A0A0H3H5K8_KLEPH	Putative transposase	4.20E-221	show
47	I5U70_33240	2548	DONE	A0A2I8DHR3_9BURK	Beta-lactamase	1.10E-191	show
48	I5U70_33245	1574	DONE	A0A0H3GZH6_KLEPH	Putative transposase	1.30E-213	show
49	I5U70_33250	31	DONE	A0A0H3GYS6_KLEPH	Transcriptional repressor protein KorC	1.20E-61	show
50	I5U70_33255	4	DONE	A0A2A4JKD7_HELVI	PX domain-containing protein	9.70E-28	show
51	I5U70_33260	228	DONE	A0A0H3GW18_KLEPH	Antirestriction protein Klca	2.10E-68	show
52	I5U70_33265	3103	DONE	W1GZE8_KLEPN	Mobile element protein	1.50E-161	show
53	I5U70_33270	11905	DONE	A0A2K8LFB0_9PROT	Transglycosylase SLT domain-containing protein	4.70E-43	show
54	I5U70_33275	0	DONE				show
55	I5U70_33280	3	DONE	N2IRB2_9PSED	Uncharacterized protein	2.00E-11	show
56	I5U70_33285	12456	DONE	A0A0D6S8T0_9PSED	Single-stranded DNA-binding protein	1.90E-53	show
57	I5U70_33290	4	DONE	A0A4P8IRX2_9BURK	Uncharacterized protein	5.80E-17	show
58	I5U70_33295	633	DONE	A0A370XAQ3_9GAMM	Uncharacterized protein	2.10E-132	show
59	I5U70_33300	12	DONE	Q9I0P4_PSEAE	Quorum threshold expression element, QteE	3.60E-39	show
61	I5U70_33305	41	DONE	N2IY70_9PSED	Uncharacterized protein	2.90E-80	show
62	I5U70_33310	289	DONE	A0A2I2KGZ7_9PROT	Uncharacterized protein	6.50E-111	show
63	I5U70_33315	70	DONE	Q3JF63_NITOC	KfrA_N domain-containing protein	2.30E-40	show
64	I5U70_33320	30	DONE	A0A1X0NA15_9PSED	Uncharacterized protein	1.60E-19	show
65	I5U70_33325	0	DONE				show
66	I5U70_33330	1	DONE	N2J025_9PSED	Uncharacterized protein	1.50E-05	show
67	I5U70_33335	0	DONE				show

68	I5U70_33340	5	DONE	A0A444X1T2_ARAHY	Uncharacterized protein	7.50E-10	show
70	I5U70_33345	5008	DONE	Q5QF62_9CAUD	Uncharacterized protein	1.20E-125	show
71	I5U70_33350	10	DONE	L7P7L6_9CAUD	Uncharacterized protein	3.70E-59	show
72	I5U70_33355	4	DONE	L7P7R7_9CAUD	Uncharacterized protein	2.50E-83	show
73	I5U70_33360	1	DONE	L7P7V3_9CAUD	Uncharacterized protein	4.20E-43	show

Table S3 ICEP33 annotation by HMMER results summary

Sequence Number	Query Name	Hits Found	Status	Top Hit			Details
				Identifier	Description	E-value	
1	I5U70_24120	5352	DONE	A9IB37_BORPD	Phage-related integrase	0.00E+00	show
2	I5U70_24125	100	DONE	A9IB36_BORPD	Uncharacterized protein	3.30E-165	show
3	I5U70_24130	1220	DONE	A9IB34_BORPD	AlpA2 protein	2.50E-41	show
4	I5U70_24135	18589	DONE	A0A1K1NNZ5_9PROT	Chromosome partitioning related protein ParA	7.00E-181	show
5	I5U70_24140	108	DONE	A9IB31_BORPD	Uncharacterized protein	1.20E-45	show
6	I5U70_24145	252	DONE	A9IB30_BORPD	Uncharacterized protein	0.00E+00	show
7	I5U70_24150	223	DONE	A0A1K1NKZ1_9PROT	Uncharacterized protein	1.60E-117	show
8	I5U70_24155	211	DONE	A0A366H727_9BURK	Uncharacterized protein	9.70E-263	show
9	I5U70_24160	224	DONE	A0A366H734_9BURK	Integrating conjugative element protein (TIGR03761 family)	9.20E-170	show
10	I5U70_24165	118	DONE	A9IB24_BORPD	Uncharacterized protein	4.90E-109	show
11	I5U70_24170	499	DONE	A0A366H8C6_9BURK	Single-strand DNA-binding protein	7.70E-99	show
12	I5U70_24175	14368	DONE	B8L445_9GAMM	DNA topoisomerase III subfamily	0.00E+00	show
13	I5U70_24180	44	DONE	A9IB14_BORPD	Uncharacterized protein	1.40E-45	show
14	I5U70_24185	118	DONE	A0A366H6R9_9BURK	Uncharacterized protein	4.60E-42	show
15	I5U70_24190	96	DONE	A0A366H6S5_9BURK	Uncharacterized protein	1.40E-84	show
16	I5U70_24195	82	DONE	A9C2Y1_DELAS	Uncharacterized protein	8.10E-159	show
17	I5U70_24200	47	DONE	A9IB02_BORPD	Uncharacterized protein	1.00E-58	show
18	I5U70_24205	96	DONE	A0A366H736_9BURK	Uncharacterized protein	1.20E-178	show
19	I5U70_24210	220	DONE	A0A366H743_9BURK	Uncharacterized protein DUF3577	2.20E-200	show

20	I5U70_24215	435	DONE	A9IAZ3_BORPD	Uncharacterized protein	5.90E-182	show
21	I5U70_24220	131	DONE	A9IAZ0_BORPD	Uncharacterized protein	2.70E-146	show
22	I5U70_24225	124	DONE	A9C2Y7_DELAS	Uncharacterized protein	1.20E-83	show
23	I5U70_24230	125	DONE	A0A1K1NKZ8_9PROT	Uncharacterized protein	4.80E-141	show
24	I5U70_24235	207	DONE	A0A1D9GZT3_9BURK	Methyltransferase	1.90E-251	show
25	I5U70_24240	317	DONE	B8L457_9GAMM	Uncharacterized protein	1.30E-61	show
26	I5U70_24245	520	DONE	A0A1D9H036_9BURK	Uncharacterized protein	2.90E-62	show
27	I5U70_24250	6651	DONE	A0A1D9GZS1_9BURK	DEAD/DEAH box helicase	0.00E+00	show
28	I5U70_24255	8	DONE	A0A1D9GZD1_9BURK	Uncharacterized protein	1.30E-09	show
29	I5U70_24260	96	DONE	A0A4R1BRR3_9PROT	Uncharacterized protein	2.80E-156	show
30	I5U70_24265	826	DONE	Q1LIW2_CUPMC	WYL domain-containing protein	2.50E-190	show
31	I5U70_24270	126	DONE	Q1LIW3_CUPMC	Uncharacterized protein	8.90E-268	show
32	I5U70_24275	387	DONE	A9BTW4_DELAS	UBA/THIF-type NAD/FAD binding protein	0.00E+00	show
33	I5U70_24280	559	DONE	A0A157SRI2_9BORD	DNA internalization-related competence protein ComEC/Rec2	3.90E-241	show
34	I5U70_24285	1	DONE	C4ZN89_THASP	Uncharacterized protein	0.0015	show
35	I5U70_24290	243	DONE	A0A1D9H002_9BURK	Integrating conjugative element protein pill, pfgi-1	1.60E-112	show
36	I5U70_24295	120	DONE	A0A366H8E9_9BURK	Uncharacterized protein	1.70E-114	show
37	I5U70_24300	246	DONE	A0A1K1NNE7_9PROT	Integrating conjugative element protein, PFL_4693 family	8.60E-150	show

38	I5U70_24305	753	DONE	A0A1K1NKW6_9PROT	Transglycosylase SLT domain-containing protein	1.50E-118	show
39	I5U70_24310	242	DONE	A9IAT3_BORPD	Conserved exported protein	1.20E-99	show
40	I5U70_24315	481	DONE	A0A366H6U5_9BURK	Conjugative coupling factor TraD (TOL family)	0.00E+00	show
41	I5U70_24320	281	DONE	A9C2Z7_DELAS	Uncharacterized protein	9.00E-164	show
42	I5U70_24325	204	DONE	A0A366H8M6_9BURK	RAQPRD family integrative conjugative element protein	5.70E-77	show
43	I5U70_24330	162	DONE	A9C2Z9_DELAS	Uncharacterized protein	6.90E-44	show
44	I5U70_24335	188	DONE	A9C300_DELAS	Uncharacterized protein	3.00E-72	show
45	I5U70_24340	180	DONE	A9IAR3_BORPD	Uncharacterized protein	5.90E-87	show
46	I5U70_24345	246	DONE	A9IAQ9_BORPD	Uncharacterized protein	3.40E-158	show
47	I5U70_24350	256	DONE	A9IAQ6_BORPD	Putative secreted protein	7.30E-200	show
48	I5U70_24355	252	DONE	A9C304_DELAS	Putative secreted protein	8.10E-307	show
49	I5U70_24360	248	DONE	A9C305_DELAS	Uncharacterized protein	2.20E-97	show
50	I5U70_24365	503	DONE	A0A366H6V6_9BURK	Conjugative transfer ATPase	0.00E+00	show
51	I5U70_24370	1551	DONE	A0A366H900_9BURK	Protein-disulfide isomerase	2.00E-153	show
52	I5U70_24375	7132	DONE	A9IAP1_BORPD	DNA repair protein radC homolog	1.90E-101	show
53	I5U70_24380	203	DONE	A9C309_DELAS	Uncharacterized protein	4.10E-88	show
54	I5U70_24385	259	DONE	A0A366H770_9BURK	Integrating conjugative element protein (TIGR03756 family)	3.00E-217	show
55	I5U70_24390	274	DONE	B8L640_9GAMM	Uncharacterized protein	7.20E-294	show
56	I5U70_24395	110	DONE	A9C312_DELAS	Uncharacterized protein	4.30E-67	show

57	I5U70_24400	308	DONE	A0A366H7Z1_9BURK	TraG-like protein	0.00E+00	show
58	I5U70_24405	105	DONE	A0A0A1VB52_9BURK	Uncharacterized protein	1.10E-77	show
59	I5U70_24410	361	DONE	A0A0A1VAC2_9BURK	Uncharacterized protein	1.50E-99	show
60	I5U70_24415	578	DONE	Q13VW0_PARXL	Transcriptional regulator, AbrB family	3.80E-59	show
61	I5U70_24420	420	DONE	B8L646_9GAMM	Relaxase	0.00E+00	show
62	I5U70_24425	32	DONE	A9C318_DELAS	Cold-shock protein DNA-binding	0.00E+00	show
63	I5U70_24430	544	DONE	B8L648_9GAMM	Uncharacterized protein	0.00E+00	show
64	I5U70_24435	21	DONE	B8L649_9GAMM	Uncharacterized protein	0.00E+00	show
65	I5U70_24440	11784	DONE	B8L650_9GAMM	AAA domain-containing protein	0.00E+00	show
66	I5U70_24445	5813	DONE	A9IAK4_BORPD	Protein kinase domain-containing protein	6.60E-305	show
67	I5U70_24450	174	DONE	A9IAK1_BORPD	Uncharacterized protein	2.80E-158	show
68	I5U70_24455	1419	DONE	A9C324_DELAS	Uncharacterized protein	4.50E-104	show
69	I5U70_24460	90	DONE	B8L653_9GAMM	Uncharacterized protein	0.00E+00	show
70	I5U70_24465	11079	DONE	B8L654_9GAMM	Putative FtsK/SpoIIIE family protein	0.00E+00	show
71	I5U70_24470	25833	DONE	A0A2P5GGI0_9ENTR	Integron integrase (Fragment)	9.20E-128	show
72	I5U70_24475	522	DONE	A0A410GDN7_9BURK	IS91 family transposase	0.00E+00	show
73	I5U70_24480	12127	DONE	A0A0H3GZW2_KLEPH	GroEL-like/integrase protein	1.30E-68	show
74	I5U70_24485	1039	DONE	W1DG09_KLEPN	Integron integrase IntI1	5.60E-62	show
75	I5U70_24490	240	DONE	A0A0D5YN51_ACIBA	2'-aminoglycoside nucleotidyltransferase AadB	6.90E-134	show
76	I5U70_24495	2296	DONE	A0A4P6VWZ2_9GAMM	Aminoglycoside N-acetyltransferase AAC(6')-IIa	7.40E-124	show

77	I5U70_24500	2710	DONE	A0A0J1GNG3_9GAMM	Beta-lactamase	3.00E-104	show
78	I5U70_24505	7683	DONE	A0A410GDQ7_9BURK	Quaternary ammonium compound efflux SMR transporter QacE delta 1	4.70E-67	show
79	I5U70_24510	8740	DONE	A0A4P6VYM7_9GAMM	Dihydropteroate synthase	1.30E-178	show
80	I5U70_24515	2321	DONE	A0A0M0HSI2_9VIBR	Acetyltransferase	1.20E-111	show
81	I5U70_24520	4	DONE	W1HRF6_KLEPN	EAL domain-containing protein	2.70E-07	show
82	I5U70_24525	654	DONE	A0A0J7XGT3_9SPHN	Transposase (Fragment)	1.60E-17	show
83	I5U70_24530	2198	DONE	Q5NUZ0_CUPMC	Hypothetical transposase for transposon Tn4378	0.00E+00	show
84	I5U70_24535	10042	DONE	Q82W59_NITEU	Site-specific recombinase	1.20E-108	show
85	I5U70_24540	42689	DONE	Q5NUV3_CUPMC	Diguanylate phosphodiesterase (EAL domain) from Tn4378(Mercury transposon)	6.50E-205	show
86	I5U70_24545	78	DONE	I9NYV9_9ALTE	Putative mercury resistance protein	2.40E-44	show
87	I5U70_24550	4321	DONE	A0A4R6R100_9BURK	MerR family transcriptional regulator	5.60E-70	show
88	I5U70_24555	52349	DONE	A0A4V3CUN8_9BURK	Mercuric reductase	0.00E+00	show
89	I5U70_24560	12012	DONE	A0A1S3JTE7_LINUN	copper-transporting ATPase 1-like isoform X5	7.70E-62	show
90	I5U70_24565	807	DONE	A0A2S0N5F9_9BURK	Mercuric transporter	4.10E-72	show
91	I5U70_24570	20937	DONE	A0A4R6R1L6_9BURK	MerR family transcriptional regulator	5.50E-90	show
92	I5U70_24575	18	DONE	A9C328_DELAS	Uncharacterized protein	1.80E-123	show
93	I5U70_24580	51	DONE	A9BLP4_DELAS	Uncharacterized protein	2.10E-52	show

94	I5U70_24585	1275	DONE	A9IAH3_BORPD	VWFA domain-containing protein	3.40E-156	show
95	I5U70_24590	5033	DONE	A0A0Q6WE99_9BURK	DEAD/DEAH box helicase	0.00E+00	show
96	I5U70_24595	2303	DONE	A0A1S1Y5Z3_9GAMM	Uncharacterized protein	2.40E-196	show
97	I5U70_24600	1991	DONE	A0A0K8QPP2_9GAMM	Uncharacterized protein	1.50E-271	show
98	I5U70_24605	6470	DONE	A0A0Q6VY80_9BURK	N-6 DNA methylase	0.00E+00	show
99	I5U70_24610	2127	DONE	A0A4R6YUC7_9GAMM	Uncharacterized protein DUF1524	0.00E+00	show
100	I5U70_24615	880	DONE	B8L661_9GAMM	WYL domain-containing protein	2.60E-195	show
101	I5U70_24620	111682	DONE	A0A1D9GZK6_9BURK	LysR family transcriptional regulator	7.60E-185	show
102	I5U70_24625	3160	DONE	A0A1I4NDC0_9PSED	Transcriptional regulator, contains XRE-family HTH domain	9.60E-58	show
103	I5U70_24630	59635	DONE	A0A1I4NDN8_9PSED	ATPase family associated with various cellular activities (AAA)	2.10E-210	show
104	I5U70_24635	490	DONE	A0A1D9HC84_9BURK	Peptidase_S8 domain-containing protein	0.00E+00	show
105	I5U70_24640	7186	DONE	A0A1D9GZH6_9BURK	Transcriptional regulator	3.20E-55	show
106	I5U70_24645	2803	DONE	A9IAE9_BORPD	Transcriptional regulator, LysR family	5.60E-118	show
